# Examining the Relationship between Park Neighborhoods, Features, Cleanliness, and Condition with Observed Weekday Park Usage and Physical Activity: A Case Study

**DOI:** 10.1155/2017/7582402

**Published:** 2017-11-26

**Authors:** Kerry Hamilton, Andrew T. Kaczynski, Melissa L. Fair, Lucie Lévesque

**Affiliations:** ^1^School of Kinesiology and Health Studies, Queen's University, 28 Division Street, Kingston, ON, Canada K7L 3N6; ^2^Department of Health Promotion, Education, and Behavior, Prevention Research Center, Arnold School of Public Health, University of South Carolina, 921 Assembly Street, Columbia, SC 29208, USA; ^3^Department of Health Promotion, Education, and Behavior, Arnold School of Public Health, University of South Carolina, 921 Assembly Street, Columbia, SC 29208, USA

## Abstract

**Background:**

Little research has comprehensively explored how park features, quality indicators, and neighborhood environments are associated with observed park usage and physical activity (PA). This case study examined whether weekday park usage and PA differ by neighborhood type, across numerous categories of park features, and according to park feature condition and cleanliness.

**Methods:**

Direct observation was used to capture the number of users and PA levels within 143 park features in 6 parks (3 urban, 3 suburban) over the course of six weeks. Audits of park environments assessed the type, condition, and cleanliness of all features and amenities.

**Results:**

Urban parks experienced greater usage, but a higher proportion of sedentary users than suburban parks. Usage and PA levels differed across types of park features, with splash pads, pools, paths, and play structures having the greatest proportion of active users. Usage did not differ by park feature condition and cleanliness, but greater condition and cleanliness were generally associated with higher PA levels.

**Conclusions:**

Factors such as neighborhood context, types of park features, and condition and cleanliness can impact park usage and PA levels and should be targets for researchers and planners aiming to foster more user-friendly and active neighborhood park environments.

## 1. Introduction

Significant health benefits of physical activity (PA) participation for all ages have been well-documented in recent decades [1–3]. These benefits include reduced risk of obesity and chronic diseases such as cardiovascular disease, diabetes, and some cancers, as well as shorter term benefits including improved psychological well-being and reduced risk of musculoskeletal problems [4, 5]. However, many Canadians are forfeiting these benefits due to insufficient PA. For example, the 2007 Canadian Health Measures Survey, using accelerometers to track moderate to vigorous PA (MVPA) in adults and children, reported only 15% of adults met the guidelines for 150 minutes of MVPA each week, and 7% of children met the guidelines for 60 minutes of MVPA each day of the week [6, 7]. The need to increase PA levels among the Canadian and worldwide populations is an escalating public health priority [8, 9].

To address these issues, researchers and public health professionals have increasingly adopted socioecological models that highlight the important role of built environment influences on PA [10, 11]. Parks, in particular, have been identified as key settings for promoting active living among children and adults given their widespread availability and low cost to use and maintain [12, 13]. However, park usage is often low and a significant proportion of park users are observed as sedentary during their visits, thus highlighting the need to increase the frequency of park visitation and PA levels of users within parks [14, 15].

A number of park characteristics have been linked to increased park usage and PA, including park proximity, size, accessibility, programming, and safety [13, 16–21]. Much research has also documented that the features of parks are vital for attracting users and providing opportunities for PA [22–24]. A variety of park facilities (e.g., playgrounds, trails, sports fields, and courts) and amenities (e.g., benches, drinking fountains, and restrooms) have been found to be associated with increased levels of PA among children and adults [25–31]. However, less research has examined the relationship between park use and PA and factors such as park quality, including condition and cleanliness, or the neighborhood environment, including geography and demography [32–34]. In one study that considered only four park features, it was found that condition of activity areas was positively associated with the number of users on basketball courts but inversely associated with the number of users and total energy expenditure in green spaces [35]. As well, Shores and West [36] reported differences in usage and PA levels between urban and rural parks but did not examine their quality or features.

To our knowledge, no published studies have comprehensively explored how the availability of numerous types of park features, the condition and cleanliness of such features, and the neighborhood environments around parks are associated with directly observed park usage and PA. Therefore, the purpose of the current paper is to present a case study examining whether weekday park usage and PA differ (1) by neighborhood type (urban versus suburban); (2) across numerous types of park features (e.g., baseball diamonds, play structures, and open fields); and (3) according to park feature condition and cleanliness.

## 2. Methods

### 2.1. Park Selection

Six parks, ranging in area from 1 to 9 hectares, from urban (*n* = 3) and suburban (*n* = 3) neighborhoods in a midsized Canadian city were included in the study. Four of these parks represented a convenience sample used in a study of park programs for families. Two additional parks with similar family appeal and neighborhood demographics were included to increase the sample size for the current study. The parks offered a wide range of facilities and amenities. Permission to observe park activity was obtained from the local Recreation & Leisure Services Department. The study was approved by the Queen's University General Research Ethics Board.

### 2.2. Neighborhood Characteristics

Table 1 presents neighborhood demographics within a 500 m buffer around each park [37]. This buffer size represents the approximate distance an average person can walk in roughly five minutes (at a speed of 5.95 km/h) and is comparable to that used in other park and PA research [34, 38]. Neighborhoods classified as urban were located closer to the downtown core of the city, had high street connectivity (i.e., grid pattern formation) and a density between 414.7 and 1057.1 dwellings per square mile, whereas suburban neighborhoods were located on the outer areas of the city, and had low street connectivity (i.e., dendrite formation) and a density between 91.9 and 262.5 dwellings per square mile. These neighborhood classifications are consistent with previous literature [39].

### 2.3. Data Collection

Data collection in parks was conducted over a 6-week period during optimal weather conditions (i.e., not raining) in the summer. Any scheduled observation time missed due to inclement weather (i.e., rain, thunderstorms, and extreme wind) or a holiday was rescheduled for the same day and time in a subsequent week. Observations were scheduled four times per day over five different days of the week and completed randomly over a 6-week period.

### 2.4. Park Usage and Physical Activity

Weekday park usage and PA levels were assessed using the System for Observing Play and Recreation in Communities (SOPARC), a valid observational tool that captures information on community recreation spaces and their users [40]. As per SOPARC protocol, each park was first strategically mapped and divided into smaller target areas [41]. These target areas segmented the park along natural boundaries and represented all standard park facilities and amenities likely to provide opportunities for park users to be physically active (e.g., green spaces, playgrounds, sport-specific fields or courts, and trails). Other park features not necessarily conducive to PA (e.g., park benches or picnic tables) were also coded as distinct target areas. A detailed map for each park was created to identify each target area as well as determine a standardized observation order. A total of 143 features and/or amenities from the six parks were identified as target areas for observation.

As per the SOPARC protocol [41], two trained observers used momentary time sampling scans to systematically record park usage and PA observations for each target area at four different time points (7:30 am, 12:30 pm, 3:30 pm, and 6:30 pm) on three different days of the week from Monday to Friday for a total of 72 hours of observation on 18 days. This observation schedule deviates slightly from the SOPARC protocol that has been validated when a weekend day is included [42]. However, it was nevertheless deemed acceptable given that data were collected during the summer vacation season. Observed park users were further categorized by sex (male and female), race/ethnicity (Latino (L), Black (B), White (W), or Others (O)), and estimated age (Child = infancy to 12 years; Teen = 13 to 20 years; Adult = 21 to 59 years; Senior = 60 years and older). Pilot testing of the protocol in the field by the two trained observers yielded 99.4% agreement on all aspects of the SOPARC tool. PA levels were recorded by categorizing the behavior of each park user according to the following definitions: Sedentary (lying down, sitting, or standing), Walking (walking or moving at a moderate pace), or Vigorous (fast-paced or high intensity activity, e.g., jogging, swinging, and doing cart wheels). These activity codes have been shown to yield high interrater agreement (i.e., 88–89.5%) [40] and criterion validity for these activity codes has been established previously through heart rate monitoring [43, 44], pedometer [45], and accelerometer [46] comparisons. Overall park usage was identified as the sum of all people within the park during the scan period, whereas target area park usage was equal to the total number of people observed on a given facility or amenity of a park during the scan period.

### 2.5. Feature Type, Condition, and Cleanliness

Type, condition, and cleanliness of the 143 features located within the six parks were assessed using the Environmental Assessment of Public Recreation Spaces (EAPRS) tool [47]. The EAPRS protocol provides a series of detailed guidelines, definitions, and visual examples to consult when conducting type, condition, and cleanliness observations. Previous research has demonstrated adequate interrater reliability and validity for the EAPRS tool [47, 48]. EAPRS observations of features were conducted on the same day that SOPARC (PA) data were collected for a given park. EAPRS observations were conducted by the first author one time, during one of the gap time periods (i.e., 8:30 am, 1:30 pm, and 4:30 pm) between SOPARC observations, within all 6 parks over a 2-week period.

Types of park features (i.e., target areas) were grouped based upon categories commonly used in the parks and PA literature [22, 49–51]. Using EAPRS classifications, ten distinct park feature types were identified: (1) paved path, (2) rough/natural path, (3) open space (open grass field or hill, treed area, and stream), (4) play structure (i.e., combination of two or more distinct features of playground equipment), (5) fields and courts (soccer, baseball, tennis, and basketball), (6) splash pad, (7) pool, (8) swing set, (9) other play features (e.g., webbed climber, slide, see saw, rock wall, and balance rockers), and (10) sitting amenities (benches, picnic tables, sheltered areas, and bleachers).

Condition and cleanliness of each of the 143 features were carefully rated as per the EAPRS protocol. Cleanliness refers to the general aesthetics and upkeep of the target area and was coded as a discrete variable categorized as 1 = not at all clean, 2 = somewhat clean, or 3 = mostly to extremely clean [47]. Condition was defined as the general state and functionality of a target area and refers to anything that might compromise the operation of the element [47]. For each target area, condition was rated as 1 = poor, 2 = fair, or 3 = excellent.

### 2.6. Analyses

Pearson's chi-square and Mann–Whitney *U* tests were used to examine associations between neighborhood type (urban versus suburban) and park usage and PA levels. Separate Kruskal-Wallis and chi-square tests were used to examine park usage and PA levels across types of park features and across condition and cleanliness ratings of park features.

## 3. Results

A total of 1098 park users were identified during the 72 observation periods. As shown in Table 2, overall park usage patterns revealed that, consistent with neighborhood demographics, most park users were female (52%) and White (89%). Children (30%) and adults (47%) used the park more frequently than teens (18%) or seniors (5%). PA recordings showed that overall, 45% of users were engaged in sedentary pursuits, 40% were walking (moderate PA), and 15% were engaged in vigorous activity. A similar proportion of users were observed during the lunch, afternoon, and evening observations (29%, 32%, and 32%, resp.), while only 6% of users were observed during the morning observation period.

### 3.1. Neighborhood Type and Park Usage and Physical Activity

Table 2 presents associations between neighborhood type (suburban versus urban) and the PA level, gender, age, race/ethnicity, and time of day of observed park users. Results from the Mann–Whitney *U* test indicated that urban neighborhoods (Median = 81.47) had higher overall park usage than suburban (Median = 54.91) neighborhoods (*U* = 1474.5, *z* = −3.68, *p* ≤ 0.001). Cramér's *V* post hoc comparisons revealed no associations between neighborhood type and the gender or race/ethnicity of park users. However, both age (*X*^2^ = 83.39, *p* < 0.001, Cramér's *V* = .28) and time of day (*X*^2^ = 20.18, *p* < 0.001, Cramér's *V* = 0.14) had a significant association with neighborhood type. Fewer teen and more adult users (13% and 52%, resp.) were seen in urban areas, whereas more teen and fewer adult users (37% and 26%, resp.) were seen in suburban areas. Urban park usage remained relatively consistent during the lunch, afternoon, and evening time points (30%, 35%, and 29%, resp., of the total urban usage), whereas suburban park usage was higher during evening observation periods (45% of the total suburban users). Chi-square test results also indicated a significant association between neighborhood type and PA (*X*^2^ = 6.402, *p* = 0.041, and Cramér's *V* = 0.07). Specifically, urban users had a greater proportion of sedentary individuals (47% of urban versus 38% of suburban) and a lower proportion of moderately (39% of urban versus 43% of suburban) and vigorously (14% of urban versus 19% of suburban) active users.

### 3.2. Park Feature Type and Usage and PA

**Figure 1:**
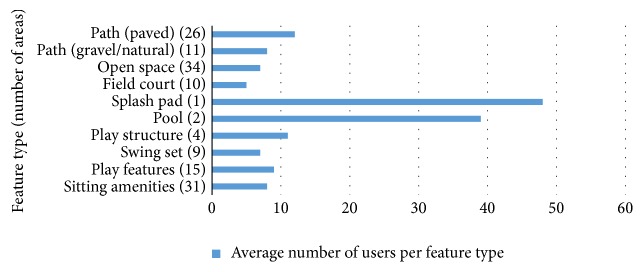


Kruskal-Wallis comparisons between feature categories found that target area park usage significantly differed across facility and amenity types (*X*_ _^2^_K-W_ = 18.48, *p* = 0.03). Statistical post hoc tests could not be performed due to a variable and small sample size between and within feature types. However, average park usage frequencies (i.e., 286 total people observed in 26 paved path target areas = 11 people per paved path area) for each feature type revealed several apparent differences. For example, both splash pad and pool showed the greatest mean usage levels, with 47 and 38 users, respectively (Figure 1). These target areas were followed by mean usage levels on paved paths and play structures, with 11 and 9.5 average users, respectively.

As shown in Table 3, there was a significant association between park feature type and PA level (*X*^2^ = 540.85, *p* ≤ 0.001, and Cramér's *V* = 0.49). Higher counts of active (moderate and vigorous) users were reported on paths (92%), open spaces (58%), splash pads (55%), and play structures (54%). Specifically, splash pads and play structures had over 20% of users categorized as vigorously active, whereas paths and open spaces had large percentages of users categorized as moderately active.

### 3.3. Park Feature Condition and Cleanliness and Usage and PA

A Kruskal-Wallis analysis comparing usage of target areas according to ratings of feature condition (*X*_ _^2^_K-W_ = 3.197, *p* = 0.20) and cleanliness (*X*_ _^2^_K-W_ = 1.140, *p* = 0.56) did not yield significant differences. However, for PA levels, as shown in Table 4, there were significant associations between feature* condition* and observed park users' PA levels (*X*^2^ = 54.94, *p* < 0.001, and Cramér's *V* = 0.154) and between* cleanliness* and PA levels (*X*^2^ = 183.24, *p* < 0.001, and Cramér's *V* = 0.290). For condition, features rated as poor, fair, and excellent had a steadily increasing proportion of users observed as vigorously active (7%, 12%, and 18%, resp.). Interestingly, the vast majority of users of features rated as poor were classified as moderately active (72%). As well, a fairly large proportion of users in fair (56%) and excellent (42%) condition areas were observed as sedentary (Table 4). With respect to cleanliness of features and PA levels, almost all users (92%) in the lowest cleanliness areas (those rated as “not at all”) and three-quarters (75%) in the “somewhat” clean areas were observed being sedentary, while two-thirds (66%) of users in the “mostly/extremely” clean areas were observed being active (moderate = 50% and vigorous = 16%).

## 4. Discussion

Parks play a vital role in facilitating PA and have become widely recognized as important resources for community health promotion [52]. However, given that PA research related to parks is still maturing, further studies are needed to determine which park and surrounding neighborhood attributes are associated with increased park usage and PA participation within parks. The analyses presented in the current paper build upon and extend prior research examining the relationship between park features and park use and PA levels to also include factors such as condition, cleanliness, and neighborhood type. A detailed case study of the specific attributes and weekday visitor behaviors within six parks provides in-depth information that can be used to inform park planning and design to promote PA and community health.

Similar to a number of other investigations reporting that up to two-thirds of park users were less than active [14, 26, 53], the present study found that almost half of park visitors were engaged in sedentary behavior. This suggests that while considerable research has shown strong associations between access or proximity to parks and greater park usage and PA levels [27, 54–56], significant potential remains to increase the energy expenditure that occurs in parks [15]. Consequently, further research is needed, via this study and others, to specifically explore which park features and other characteristics are related to use of, and activity within, parks [25, 35]. Such insights can provide guidance to park planners and neighborhood developers with respect to the design and maintenance of parks for facilitating increased park usage, PA levels, and community health.

### 4.1. Neighborhood Type

The current study was unique in its exploration of differences between urban and suburban park usage and PA. Parks in urban neighborhoods had a significantly greater median number of users, which contrasts somewhat with a previous study in which urban parks had lower numbers of total users than parks in more rural areas [36]. However, despite a greater level of park usage, the present study also found that urban parks had a higher proportion of sedentary users and lower levels of both moderate and vigorous PA than suburban parks, which is comparable to a study reporting that urban park users were more physically active than rural visitors [36]. We also found that park use varied throughout the day according to neighborhood type: urban park usage was distributed fairly evenly across lunch, afternoon, and evening observation periods (although considerably lower during the mornings), whereas suburban park use was weighted more heavily toward the evening hours.

These findings indicate that further research is needed to explore the differences in park usage and PA levels by neighborhood type and rurality. Specifically, research should parse out how patterns of use vary throughout the day across the park and what specific factors (e.g., playgrounds, restrooms, and shade) are associated with park use and PA levels by all three neighborhood types (i.e., rural, suburban, and urban). Optimal park design would, of course, attempt to maximize park usage and activity levels of visitors throughout the day while minimizing crowding and conflicts among distinct visitor groups, but more research is needed to inform such park planning considerations in diverse geographic settings.

This study suggests that there may be benefits of urban neighborhood designs over suburban designs in promoting higher levels of park usage. One possible explanation is that urban environments typically provide more walkable environments [57, 58] with more destinations around parks [59, 60]. Thus, it may be simply that urban parks can be used as both a recreational destination and a utilitarian connection (i.e., to get from point A to point B), whereas suburban parks are more likely to be only a recreation destination. Shores and West [36] also found that rural parks, which are similar to suburban parks in that they do not typically provide active connections to other destinations, were mainly used during evening hours and for recreation leisure activities. This suggests that suburban parks may need to rely more on internal park characteristics to entice people to visit the park and be active within it, whereas urban neighborhoods can rely on other resources in the surrounding environment to encourage park usage. In light of accumulating evidence about the association between the neighborhood built environment and its active use for population health benefits, this study highlights that neighborhood type is an important park characteristic to be considered [34, 61–63].

At the same time, the higher number of urban park users engaged in lower levels of PA merits some concern and suggests that while providing parks close to urban areas that are daytime workforce and destination hubs may increase park use, additional research into designing and planning urban parks that facilitate increased PA is warranted. This finding is of particular interest in light of the difference in household income between the two neighborhood types, with urban parks being located in areas with significantly lower socioeconomic status. Typically, less affluent individuals exhibit lesser leisure-time PA participation [64] and while evidence is mixed on whether lower socioeconomic status (SES) neighborhoods possess fewer parks overall, most research indicates that the parks in such areas are of poorer quality and have fewer features and amenities and lesser access to programming, which likely contributes to lower levels of observed park-based PA in such contexts [65–69]. Further research is needed to explore how type and quality of amenities and programming are related to PA levels based not only on neighborhood type, but also on neighborhood SES.

Finally, while the present study took into account the crude neighborhood type (urban versus suburban) in relation to usage and PA levels within parks, it did not account for specific neighborhood features that may influence these behaviors. For example, a study of 20 parks located in the US and Belgium found that greater neighborhood walkability was associated with increased park use [70]. Somewhat in contrast, Kaczynski et al. [34] reported that greater mixed land use around parks was related to lesser odds of them being used for PA. The association between neighborhood context and park use and PA in both urban and rural contexts may be influenced by specific features that facilitate these behaviors. For example, one study reported that greater street connectivity and not having to cross or travel on a high traffic speed road were associated with a greater likelihood of using parks and park-based PA [20]. In general, more research is needed that builds upon the present study examining differences in neighborhood type, while also examining the influence of diverse neighborhood characteristics.

### 4.2. Feature Type

The findings from the current study show that, in addition to external (i.e., neighborhood) park characteristics, internal features (e.g., facilities and amenities) also play a role in influencing park usage and PA levels. For example, paved walking paths, play structures, and water features (splash pads and pools) had the highest numbers of observed users. Consistent with numerous prior studies, walking trails, both paved and natural, facilitated the highest proportion of PA, being used almost exclusively for moderate and vigorous activity [22, 71]. Also, over half of park users observed on play structures, splash pads, and open spaces were engaged in moderate to vigorous activity [22, 50, 51, 72]. Also of note, was that while play structures (i.e., playgrounds) were significantly associated with higher levels of moderate to vigorous PA, other play features such as rock walls, seesaws, and webbed climbers were used for sedentary activity by two-thirds of users, and swings were divided into their ability to facilitate PA with over half of users engaged in sedentary activity and the majority of the remaining users in vigorous activity. Prior research observing park play features has also yielded inconsistent results, with some studies indicating play structures are positively associated with PA, while others have found no influence and very few studies have individually examined specific play features (i.e., swings versus slides) and their influence on PA levels [73–75]. Therefore, while these play features may be aesthetically appealing and are commonly incorporated into park design, further research is needed to determine their distinct abilities to encourage active play among youth.

These findings related to feature type, in conjunction with neighborhood type, could be used by municipalities striving to promote PA among community members by optimizing the availability and accessibility of specific park features and programs that reflect the needs of the surrounding neighborhood demographics and those of actual parks users, while also considering patterns of park usage (i.e., time of day most frequently utilized). For example, municipalities could better connect and provide neighborhood maps illustrating walkable routes between local parks and other neighborhood destinations in suburban neighborhoods or offer improved walking trails for adults in urban neighborhoods [17, 76]. Additionally, offering and promoting suburban park programs for young families during evening hours and for urban teens during late afternoon hours could facilitate increased park usage. Studies such as this one that examine specific visitors and their usage patterns as well as the park attributes that are associated with greater PA can continue to cultivate the PA-promoting potential of parks.

### 4.3. Cleanliness and Condition

The current study is one of the first attempts to relate condition and cleanliness ratings of several neighborhood park features to observed target area usage and PA levels. Greater park feature cleanliness and condition were not significantly associated with increased usage, whereas previous studies have reported that issues such as the presence of litter, dirty play structures and surfaces, lack of grass, and damaged sidewalks can negatively affect park use [12, 21, 51]. While these findings were not significant for park use, improved park feature condition and cleanliness were associated with an increased in number of users engaged in vigorous PA, thus providing promising evidence to support the growing body of research on the impact of park quality on PA [21, 32, 73]. Previous observational studies have had difficulty quantifying the implications of this association or have found conflicting evidence on the impacts of condition and cleanliness by feature type [35, 73]. Therefore, further studies similar to the present one are needed to continue to better understand the relationship between park condition and cleanliness and its impact on PA levels of park users.

Interestingly, the same findings were not present for moderately active users, with a high proportion of visitors in poor condition target areas engaging in moderate PA, while a large percentage of park users exposed to fair and excellent condition features were still observed engaging in sedentary behavior. This observed difference in PA could be due in part to the variation in the type and number of park features and the condition and cleanliness of said features across urban versus suburban parks, which the present study did not account for. Future research examining the relationship between PA levels and cleanliness and condition by neighborhood type (i.e., urban versus rural), while also accounting for variation of number and type of features, would be of use in determining which factor is the stronger predictor of PA. Doing so would help park managers efficiently allocate resources toward feature offerings, maintenance of those features, or both to maximize the potential of parks to foster PA.

### 4.4. Limitations

Although the six parks in this study shared similar park characteristics, parks in general tend to contain a wide variety of features that lend themselves to different types of usage [12, 77]. The SOPARC tool uses these features, instead of individuals, as the units of analysis [40]. Consequently, the variability of park features, coupled with a small park sample size, limited some of the analyses that could be conducted. Although this study is similar to other time-intensive SOPARC researches that have included between 4 and 8 parks [14, 50, 53, 78], similar studies with larger sample sizes would allow more comprehensive analyses of diverse neighborhood populations and attributes, park features and amenities, and other park attributes (e.g., quality, safety). Also, due to limited resources and time, weekend observations were not collected within the context of this study. Although this is not consistent with usual SOPARC protocols [79], it is likely that any differences between weekday and weekend usage were minimized by the summer data collection time period because many Canadian families are vacationing out of town on weekends and/or are taking time off during the weekdays in the temperate summer months. This study otherwise employed an observation plan resembling the suggested schedule of 4 days/week, 4 times/day determined to be sufficient for obtaining a robust description of park usage and PA levels [42]. Moreover, with the exception of certain results for gender (i.e., more female users observed) and field and court usage (i.e., less field and court usage observed), our findings mirror similar studies using a full 7-day park observation protocol [31, 40, 49, 79]. Nevertheless, the absence of a weekend day as part of our data collection protocol may have skewed some of our findings if, for example, parks in certain neighborhoods (e.g., suburban versus urban) or with particular features (e.g., paths, play features, and sitting amenities) experience differential user characteristics or PA levels on Saturdays or Sundays. Additionally, since SOPARC utilizes momentary time sampling, this method is unable to assess duration of PA and only has three broad categories of PA levels that can be used to estimate energy expenditure, thereby making detailed analyses of PA expenditure difficult [40].

Lastly, the present study provided novel comparisons across neighborhood types for park use and PA but did not consider how such behaviors differ when also accounting for the variation of type, condition, and cleanliness of park features. This is of interest given that prior research has indicated that lower income communities, often located in urban areas, frequently have poorer quality and fewer park features when compared to higher wealth neighborhoods [65, 69].

## 5. Conclusion

Findings from this study indicate that target area park usage differs according to park feature types and neighborhood types, with the most notable differences seen in urban neighborhoods and on features such as splash pads, pools, paths, and play structures. Levels of actual PA in parks were generally associated with park feature type, condition and cleanliness, and neighborhood type. As PA research related to parks is still maturing, this study makes an important contribution to furthering our understanding of the role of park feature type, condition, cleanliness, and neighborhood type in facilitating park usage and PA levels. Findings from this study can guide future researchers, practitioners, planners, and designers in promoting and creating more user-friendly and active neighborhood park environments.

## Figures and Tables

**Table 1:** Neighborhood demographics within a 500 m radius around each study park.

Neighborhood characteristic (2006 census)	Neighborhood type					
	Suburban			Urban		
	Park 1	Park 2^a^	Park 3	Park 4	Park 5	Park 6
Total population (*N*)	5,835	1,982	5,339	11,279	10,905	7,503
Gender						
Female	51%	51%	54%	52%	52%	54%
Male	49%	49%	46%	48%	48%	46%
Age						
Child	12%	18%	11%	7%	7%	15%
Teen	19%	17%	13%	8%	11%	14%
Adult	65%	56%	54%	69%	68%	56%
Senior	21%	9%	21%	19%	21%	18%
Ethnicity						
White	93%	95%	93%	92%	89%	94%
Others	7%	5%	7%	8%	11%	6%
Land area dwellings (/mile^2^)	262.5	204.4	91.9	1057.3	975.1	414.7
Mean household income ($)	100,381	104,547	72,102	44,435	51,959	39,110
Education^b^ (%)	86%	64%	82%	88%	81%	84%

^a^Neighborhood was not fully developed at the time of census 2006 collection. ^b^Education = total population completed a high school certificate, diploma, or a degree.

**Table 2:** Associations between neighborhood type and park usage and physical activity.

Variable category	Total (*N*)	Neighborhood type				*X* ^2^	*p*
		Suburban		Urban			
		*N*	(%)	*N*	(%)		
Physical activity							
Sedentary	490	88	(38)	402	(47)	6.40	0.041
Moderate	438	100	(43)	338	(39)		
Vigorous	163	43	(19)	120	(14)		
Gender							
Female	565	120	(53)	445	(51)	0.84	0.772
Male	526	104	(47)	422	(49)		
Age							
Child	333	73	(32)	260	(30)	83.39	<0.001
Teen	195	84	(37)	111	(13)		
Adult	514	61	(26)	453	(52)		
Senior	54	12	(5)	42	(5)		
Race/ethnicity							
White	962	200	(87)	762	(89)	1.03	0.310
Others	120	30	(13)	90	(11)		
Time of day							
Morning	67	12	(5)	55	(6)	20.18	<0.001
Lunch	318	58	(25)	260	(30)		
Afternoon	357	58	(25)	299	(35)		
Evening	356	103	(45)	253	(29)		

**Table 3:** Pearson chi-square associations between target area type and physical activity levels.

Target area type	Physical activity level						*X* ^2^	*p*
	Sedentary		Moderate		Vigorous			
	*n*	(%)	*n*	(%)	*n*	(%)		
Path (paved)	22	(7.7)	207	(72.6)	56	(19.6)	540.85	0.00^*∗∗*^
Path (gravel + natural)	7	(8.0)	69	(79.3)	11	(12.6)		
Open space	77	(41.8)	92	(50.0)	15	(8.2)		
Field or court	25	(65.8)	6	(15.8)	7	(18.4)		
Splash pad	21	(44.7)	15	(31.9)	11	(23.4)		
Pool	46	(60.5)	18	(23.7)	12	(15.8)		
Play structure	16	(45.7)	11	(31.4)	8	(22.9)		
Swing set	28	(53.8)	2	(3.8)	22	(42.3)		
Play features	64	(66.0)	15	(15.4)	18	(18.5)		
Sitting amenities^a^	184	(97.4)	2	(1.1)	3	(1.6)		

^a^Picnic table, benches, bleachers, and sheltered areas; ^*∗∗*^*p* < 0.001.

**Table 4:** Associations between park feature and amenity condition and cleanliness and physical activity.

Feature and amenity ranking	Physical activity observations						*X* ^2^	*p*
	Sedentary		Moderate		Vigorous			
	*n* = 490	(%)	*n* = 438	(%)	*n* = 163	(%)		
Condition *n* = 143							54.941	0.000^*∗∗*^
Poor	18	(21)	60	(72)	6	(7)		
Fair	191	(56)	111	(32)	40	(12)		
Excellent	281	(42)	267	(40)	117	(18)		
Cleanliness *n* = 143							183.24	0.000^*∗∗*^
Not at all	56	(92)	4	(7)	1	(1)		
Somewhat	160	(75)	26	(12)	28	(13)		
Mostly/extremely	274	(34)	408	(50)	134	(16)		

*Note*. Value indicates number of people observed within each category. Frequency percentages reflect condition and cleanliness rating between each physical activity level; ^*∗∗*^*p* < 0.001.
